# Knowledge, attitude, and practice of oral nutritional supplements in patients undergoing radiotherapy for head-and-neck cancer

**DOI:** 10.3389/fnut.2025.1633423

**Published:** 2025-10-22

**Authors:** Yajun Chen, Shuang Yang, Yuying Li, Chunlei Li, Mengyuan Chen, Ruina Wang, Na Liu, Yu Fang, Mingming Han, Hongwei Tang, Jun Zhang, Yaqi Zeng, Yueying Li, Yujie Wang, Ximei Zhang, Peiguo Wang, Kun Wang

**Affiliations:** ^1^Department of Nutrition, Key Laboratory of Cancer Prevention and Therapy, Tianjin Medical University Cancer Institute and Hospital, National Clinical Research Center for Cancer, Tianjin’s Clinical Research Center for Cancer, Tianjin, China; ^2^Department of Radiotherapy, Key Laboratory of Cancer Prevention and Therapy, Tianjin Medical University Cancer Institute and Hospital, National Clinical Research Center for Cancer, Tianjin’s Clinical Research Center for Cancer, Tianjin, China; ^3^Department of Nutrition, Beijing Cancer Hospital, Beijing, China; ^4^Department of Nutrition, Tianjin Medical University General Hospital, Tianjin, China; ^5^Department of Nutrition, Wuqing People’s Hospital, Tianjin, China; ^6^Department of Nutrition, Tianjin Union Medical Center, Tianjin, China

**Keywords:** head-and-neck cancer, oral nutritional supplements, knowledge, attitude, practice

## Abstract

**Background:**

This study aimed to explore the knowledge, attitude, and practice (KAP) of oral nutritional supplementation in patients undergoing radiotherapy for head-and-neck cancer.

**Materials and methods:**

A multicenter cross-sectional study was conducted from December 2023 to July 2024 across five medical institutes. Demographic data and KAP scores were collected and assessed using a self-developed validated questionnaire. A threshold of ≥70.0% of the maximal scores was established to define good knowledge, a positive attitude, and active practice.

**Results:**

A total of 437 valid questionnaires were analyzed; the majority of participants were men (70.71%) and aged between 41 and 60 years (50.11%). The median knowledge score was 4 (range: 0–10; possible range: 0–20), the median attitude score was 34 (range: 30–39; possible range: 10–50), and the median practice score was 15 (range: 13–17; possible range: 4–20). The knowledge score exhibited a positive correlation between both attitude (r = 0.379, *p* < 0.001) and practice (r = 0.395, *p* < 0.001) scores; attitude and practice scores were also positively correlated (r = 0.363, *p* < 0.001). Structural equation modeling showed that knowledge had a direct positive effect on attitude (*β* = 0.613, *p* < 0.001) and practice (β = 0.807, *p* < 0.001), while attitude had a direct effect on practice (β = 0.614, *p* < 0.001).

**Conclusion:**

The findings indicate that patients with head-and-neck cancer undergoing radiotherapy exhibit poor knowledge and unfavorable attitudes toward oral nutritional supplements, despite displaying proactive practices. Considering the positive correlation among KAP, the provision of personalized medical education is essential for effective disease management.

## Introduction

Head-and-neck cancer (HNC) is a group of malignant tumors with more than 660,000 new cases worldwide, 325,000 deaths annually, and incidence rates gradually increasing over the past decades (1). In the majority of cases, HNC development results in a decrease in nutrient intake, significant weight loss, and a decline in muscle mass, weakening the patient’s overall health, as well as increasing the likelihood of treatment complications (2, 3). Considered as a treatment choice in treating HNC, radiotherapy often leads to side effects such as difficulty swallowing, taste changes, and mucositis, which can further impair food intake and worsen cancer-related malnutrition (4). As a result, worsening of nutritional status is associated with reduced treatment tolerance, increased infection risk, and slower recovery, impacting patients’ quality of life and survival rates (5). Proactive nutritional assessment and support are essential to optimize treatment effectiveness and improve resilience during radiotherapy.

Patients undergoing radiotherapy, particularly those with HNC, were shown to greatly benefit from nutritional counseling and supplementation, which could reduce the treatment-related side effects of malnutrition (6). Although a number of studies have shown that oral nutritional supplements significantly improve the symptoms of patients undergoing radiotherapy for HNC (6–8), patients’ awareness and practice are still lacking. Many HNC patients show very low awareness of oral nutritional supplementation; this issue is widespread across different regions and healthcare systems, underscoring the urgent need for comprehensive global support mechanisms to address these gaps (9, 10). Similarly, the use of nutritional supplements among Chinese patients has been reported to be insufficient, with patients demonstrating limited awareness of the benefits and importance of oral nutritional supplementation, leading to inconsistent practices (11). This variation in understanding can result in suboptimal nutritional intake, further exacerbating malnutrition risks during treatment.

Knowledge, attitude, and practice (KAP) assessment could be used as a tool to increase the understanding of patients toward specific healthcare interventions or uncover the barriers in the implementation of such interventions (12, 13). Previous studies assessing KAP toward nutrition supplementation and healthy eating reported many challenges, uncertainty, confusion, and isolation among HNC patients, with the notable need for improvements (14–16). Currently, there is no study evaluating KAP of oral nutritional supplements among Chinese HNC patients after radiotherapy.

This study aimed to explore the current status of knowledge, attitude, and practice of oral nutritional supplementation in patients with HNC undergoing radiotherapy.

## Methods

### Study design and patients

This multicenter cross-sectional KAP study was conducted on HNC patients who underwent radiotherapy between December 2023 and July 2024 in China and carried out in the radiotherapy and oncology departments of Tianjin Medical University Cancer Institute and Hospital and four other medical institutes—Peking University Cancer Hospital, Tianjin Medical University General Hospital, Tianjin People’s Hospital, and Tianjin Wuqing District People’s Hospital. The study has been approved by the Medical Ethics Committee of Tianjin Medical University Cancer Institute and Hospital (approval number: EK2023229), and informed consent was obtained from the study subjects.

The inclusion criteria were as follows: (1) patients diagnosed with HNC who were scheduled to undergo radiotherapy or had already received radiotherapy; (2) those aged 18 years and above; and (3) those able to understand the purpose of the study and who voluntarily signed informed consent. No specific exclusion criterion was applied in the study.

### Questionnaire

The design of the questionnaire referred to previous studies on oral nutritional supplements in HNC patients receiving radiotherapy (7, 8) and relevant guidelines, including the *Radiation Therapy Guidelines for HNC in China (2021)* and the *Expert Consensus on Oral Nutritional Supplementation for Radiation Therapy Patients with Oncology* (2017) (17, 18). After the completion of the first draft, the opinions of five experts in oncology and nutrition were taken into account to confirm the content validity, and the questionnaire was revised according to the experts’ suggestions. After the completion of the pilot investigation (31 cases), the reliability was tested, and the overall Cronbach’s *α* coefficient was 0.935. The Cronbach’s α coefficient was 0.971 for the knowledge dimension, 0.916 for the attitude dimension, and 0.762 for the practice dimension. The internal consistency of the questionnaire was therefore deemed acceptable. During the pilot study, participants were also asked about the clarity and relevance of the items. No issues of ambiguity or redundancy were reported, supporting the face validity of the questionnaire.

The final questionnaire was in Chinese, and the content of the questionnaire included four aspects: demographic information, knowledge dimension, attitude dimension, and practice dimension. The demographic data included age, gender, residence, education level, marital status, monthly household income per capita, height, weight, tumor location, time of diagnosis, and treatments. The body mass index (BMI) is determined by dividing weight in kilograms by the square of height in meters. The BMI thresholds were based on the Chinese classification system, which categorizes individuals as underweight (<18.5 kg/m2), normal weight (18.5 to <24 kg/m2), overweight (24 to <28 kg/m2), and obesity (≥28 kg/m2) (19).

The knowledge dimension consisted of 10 items. The score ranged from 0 to 20, with 2 points assigned for a “very familiar” response, 1 point for “heard about,” and 0 points for “unclear.” The attitude dimension consisted of 10 questions measured on a five-point Likert scale, with responses ranging from strongly agree to strongly disagree (5–1), resulting in total scores from 10 to 50. The practice dimension included four questions, with responses scored from 1 to 5, ranging from never to always, for a total score between 4 and 20 points. Questions 5 and 6 in the practice section, which assessed issues encountered during the use of oral nutritional supplements and sources of knowledge about oral nutrition supplementation, were not scored and were analyzed descriptively. The threshold of good knowledge, positive attitude, and active behavior was considered to be ≥70.0% (20).

### Questionnaire distribution and quality control

The questionnaire was distributed to eligible participants in the departments of radiotherapy and oncology and in the outpatient ward. A convenience sampling method was used, and the questionnaire was distributed in both electronic (online) and paper (offline) versions. The electronic questionnaire was answered by scanning the code in the hospital. Dedicated researchers helped to interpret the contents of the questionnaire for the patients if they had questions. Special checks were performed for distribution and retrieval, data entry, and inspection.

### Sample size

The sample size was calculated based on the item-to-response ratio of 1:15 recommended for questionnaire research (21). With 24 KAP items in the questionnaire (excluding demographics and two descriptive questions), the required sample size was 360. Considering a 15% dropout rate, the minimal sample size was 414.

### Statistical methods

Statistical analysis was performed using SPSS 27.0 (IBM, Armonk, NY, USA) and AMOS 26.0 (IBM, Armonk, NY, USA). The distribution of the scores of each dimension was tested for data normality. If a skewed distribution was observed, the data were expressed as medians, 25% quantiles, and 75% quantiles. The Wilcoxon–Mann–Whitney U-test or the Kruskal–Wallis analysis of variance was used for comparison. Enumeration data for different demographic characteristics and responses to each question were expressed as N (%). Correlation analysis was performed using Spearman’s correlation coefficient. Combined with the KAP theoretical framework, the structural equation model (SEM) was used to verify whether attitude plays a mediating role between knowledge and practice, and the sizes of the indirect and direct effects were calculated and compared. The hypotheses tested were as follows: (H1) knowledge directly affects attitude, (H2) knowledge directly affects practice, and (H3) knowledge indirectly affects practice through attitude. The threshold criteria for the goodness-of-fit indices of the corresponding SEM model were as follows: root mean square error of approximation (RMSEA) < 0.08, standardized root mean squared residual (SRMR) < 0.08, comparative fit index (CFI) > 0.8, and Tucker–Lewis index (TLI) > 0.8. Two-sided *p*-values of < 0.05 were considered to indicate statistical significance for all analyses.

## Results

### General characteristics of participants

A total of 454 questionnaires were collected. Two questionnaires from participants under 18 years of age were excluded, along with one questionnaire that contained logical errors regarding the type of medical insurance. Four questionnaires with incomplete responses and 10 questionnaires with implausible BMI values were also excluded. Ultimately, 437 valid questionnaires were included in the analysis, resulting in an effective response rate of 96.26%. The majority of participants were men (70.71%), married (91.30%), urban residents (50.80%), and aged 41–60 (50.11%) years. The most common cancer type was nasopharyngeal carcinoma in 37.76% of participants, followed by oral cancer in 20.14% and laryngeal cancer in 11.90%. Approximately one-third (34.78%) of participants received surgical treatment, 62.02% underwent chemotherapy, 9.61% underwent targeted therapy, and 13.73% underwent immunotherapy. Only 8.92% of participants were underweight (BMI < 18.5). Moreover, only 27.0% of participants reported receiving educational information about oral nutritional supplements in the hospital (Table 1).

**Table 1:** General characterization of the study participants.

Characterization	*N* (%)	Knowledge		Attitude		Practice	
		M (Q_1_, Q_3_)	*p*	M (Q_1_, Q_3_)	*p*	M (Q_1_, Q_3_)	*p*
Total	437	4 (0, 10)		34 (30, 39)		15 (13, 17)	
Gender			0.291		0.920		0.672
Men	309 (70.71)	3 (0, 10)		34 (31, 39)		15 (13, 17)	
Women	128 (29.29)	4.5 (0, 10)		35 (30, 39.75)		15 (13, 17)	
Age (years)			0.383		0.092		0.035
18 to 40	51 (11.67)	5 (1, 10)		37 (32, 40)		16 (14, 17)	
41–60	219 (50.11)	4 (0, 10)		33 (30, 39)		15 (13, 17)	
>60	167 (38.22)	3 (0, 9)		34 (30, 40)		14 (13, 17)	
Place of residence			0.016		0.443		0.105
Rural areas	215 (49.20)	3 (0, 9)		34 (30, 40)		15 (13, 16)	
Cities	222 (50.80)	5 (0, 10)		35 (30, 39)		15 (13, 18)	
Degree of education			0.003		0.029		0.011
Junior high school and below	238 (54.46)	3 (0, 9)		33 (30, 40)		15 (13, 16)	
High school/technical secondary school	117 (26.77)	5 (0, 10)		34 (31, 39)		15 (13, 17)	
Junior college	36 (8.24)	4 (0, 9)		34.5 (31, 38)		16 (14, 18)	
Bachelor degree or above	46 (10.53)	7.5 (2, 12)		38 (33.5, 40)		16 (14, 19)	
Marriage			0.751		0.486		0.288
Married	399 (91.30)	4 (0, 10)		34 (30, 39)		15 (13, 17)	
Other	38 (8.70)	4.5 (1, 9.25)		35 (31, 40)		15 (13, 16)	
Monthly household income per capita (Yuan)			0.020		0.003		0.008
<2,000	113 (25.86)	2 (0, 7)		33 (30, 39)		14 (12, 16)	
2,000–5,000.	229 (52.40)	4 (0, 10)		33 (30, 39)		15 (13, 17)	
5,000–10,000.	75 (17.16)	6 (0, 10)		37 (33, 40)		16 (14, 17)	
>10,000	20 (4.58)	7 (0.5, 13.75)		38.5 (35.25, 40)		14.5 (12.25, 18.5)	
The location of primary tumor			0.065		0.079		0.003
Nasopharyngeal carcinoma	165 (37.76)	3 (0, 9.5)		33 (30, 38.5)		15 (13, 17)	
Laryngeal cancer	52 (11.90)	3.5 (0, 10)		34 (30, 40)		14 (12, 16)	
Oral cancer	88 (20.14)	5.5 (1, 10)		35.5 (32, 40)		16 (13.25, 19)	
Pharyngeal carcinoma	40 (9.15)	3 (0.25, 7.75)		33 (31.25, 38)		14 (13, 16)	
Other	92 (21.05)	5 (0, 9)		35 (30, 40)		15 (13, 17)	
Time of diagnosis			0.052		0.096		0.104
Within 1 month	69 (15.79)	1 (0, 7)		32 (30, 39)		14 (13, 16)	
1–3 months	155 (35.47)	3 (0, 9)		33 (30, 39)		15 (13, 17)	
4–6 months	98 (22.43)	4 (0.75, 10)		35 (31, 40)		15.5 (13.75, 17)	
7 months–1 year	43 (9.84)	5 (0, 10)		35 (31, 40)		16 (13, 18)	
More than 1 year	72 (16.48)	6 (1, 10)		35 (31.25, 39)		15.5 (13.25, 17)	
The number of radiotherapy treatments			<0.001		0.004		0.010
Not yet started	192 (43.94)	3 (0, 8)		33 (30, 39)		15 (13, 16)	
Less than 10 times	128 (29.29)	3 (0, 10)		34 (30, 39)		15 (13, 17)	
11–20 times	39 (8.92)	3 (0, 10)		35 (31, 38)		16 (13, 16)	
21–30 times	40 (9.15)	7 (3, 11.75)		37 (31.25, 40)		16 (14.25, 19)	
31 to 40 times	38 (8.70)	9 (2.75, 13)		38 (33, 40)		16 (14, 18)	
Received surgical treatment			0.001		0.023		<0.001
Yes	152 (34.78)	6 (1, 10)		35 (31, 40)		16 (14, 18)	
No	285 (65.22)	3 (0, 9)		33 (30, 39)		15 (13, 16)	
Received chemotherapy			0.022		0.036		0.035
Yes	271 (62.01)	4 (0, 10)		35 (31, 39)		15 (13, 17)	
No	166 (37.99)	2.5 (0, 10)		32 (30, 40)		14 (13, 16.25)	
Received targeted therapy			0.023		0.271		0.491
Yes	42 (9.61)	7.5 (1.75, 10)		35.5 (32, 39)		15.5 (13, 17.25)	
No	395 (90.39)	4 (0, 9)		34 (30, 40)		15 (13, 17)	
Received immunotherapy			0.918		0.629		0.361
Yes	60 (13.73)	4 (0, 10)		35 (31, 38.75)		15.5 (14, 17)	
No	377 (86.27)	4 (0, 10)		34 (30, 40)		15 (13, 17)	
BMI							
<18.5	39 (8.92)	6 (1, 13)		35 (31, 40)		15 (13, 18)	
18.5–23.9	225 (51.49)	4 (0, 10)		34 (30, 40)		15 (13, 17)	
24.0 or higher	173 (39.59)	4 (0, 9)		34 (30, 39)		15 (13, 17)	
Medical education on oral nutritional supplements			<0.001		<0.001		<0.001
Yes	118 (27.00)	9 (3, 11.25)		35 (31, 40)		16 (14, 19.25)	
No	319 (73.00)	2 (0, 7)		34 (30, 39)		14 (13, 16)	

The “others” category includes tongue cancer, tonsillar cancer, gingival cancer, malignant tumors of the maxilla and mandible, parotid gland cancer, and hypopharyngeal cancer.

### Influencing factors of the KAP score

Knowledge scores were significantly lower among participants from rural areas (*p* = 0.016); those with lower education levels (*p* = 0.003); those with lower income (*p* = 0.020); those who underwent fewer radiotherapy sessions (*p* < 0.001); and those who had not received surgical treatment (*p* = 0.001), radiotherapy (p = 0.003), chemotherapy (*p* = 0.022), or targeted therapy (*p* = 0.023). Scores were most notably lower among participants who had never received any educational information about oral nutritional supplements in the hospital (p < 0.001).

Attitude scores were significantly lower among participants with lower education (*p* = 0.029), lower income (*p* = 0.003), fewer radiotherapy sessions (*p* = 0.004), and those who had not received surgical treatment (*p* = 0.023), radiotherapy (*p* = 0.017), or chemotherapy (*p* = 0.036). Scores were also significantly lower in participants who had never received any educational information about oral nutritional supplements in the hospital (*p* < 0.001). Practice scores were significantly lower among older participants (*p* = 0.035); those with lower education levels (*p* = 0.011), lower income (*p* = 0.008), or laryngeal/pharyngeal cancer (*p* = 0.003); and those who had undergone fewer radiotherapy sessions (*p* = 0.010). Scores were also lower among participants who had not received surgical treatment (*p* < 0.001), radiotherapy (p = 0.003), or chemotherapy (*p* = 0.035). Additionally, practice scores were significantly lower in participants who never received any educational information about oral nutritional supplements in the hospital (p < 0.001). Detailed information on participants and their KAP scores is shown in Table 1.

### Analysis of the responses to the KAP questions

The median knowledge score in this study was 4 (0, 10), corresponding to a poor knowledge level. Questions unclear to the majority of respondents included the oral nutritional supplements lowering total medical costs (question K10, unclear to 70.02%, with 22.43% not sure), reducing complications (question K9, unclear to 61.56%, with 29.75% not sure), and the exact way of taking supplements (question K8, unclear to 62.93%, with 27.69% not sure) (Table 2).

**Table 2:** Distribution of scores in the knowledge dimensions.

*N* (%)	Very familiar	Have heard of	Not clear
1. Oral nutritional supplement (ORNS) is a preparation that provides a variety of macronutrients and micronutrients in liquid, semi-solid, or powder form and is added to drinks or foods for oral use for the purpose of increasing oral nutrient intake.	55 (12.59)	200 (45.77)	182 (41.65)
2. Oral nutritional supplements can be divided into either total-nutrient or non-total-nutrient forms. Total-nutrient forms can be used as the sole source of long-term nutrition.	43 (9.84)	137 (31.35)	257 (58.81)
Oral nutritional supplements are the first choice of medical nutritional supplement recommended by the guidelines.	43 (9.84)	144 (32.95)	250 (57.21)
4. Oral nutritional supplements include meal supplement (extra meal), partial meal replacement (meal), and whole meal replacement (meal replacement, full amount, and multiple meals).	47 (10.76)	160 (36.61)	230 (52.63)
5. Oral nutritional supplement is a balanced formula, which contains the three major nutrients and various minerals and vitamins needed by the human body.	48 (10.98)	173 (39.59)	216 (49.43)
6. Oral nutritional supplements are applicable to a wide range of patients, including all types of hospitalized patients with malnutrition or nutritional risk and some patients with malignant tumors undergoing surgery or radiotherapy and chemotherapy.	48 (10.98)	173 (39.59)	216 (49.43)
7. Oral nutritional supplements can help alleviate malnutrition during radiotherapy for head-and-neck cancer.	46 (10.53)	169 (38.67)	222 (50.8)
8. When taking oral nutritional supplements in the early stage, sip slowly and start with a small amount; otherwise, the intestine will not adapt to the high concentration of nutritional preparations, which may cause diarrhea.	41 (9.38)	121 (27.69)	275 (62.93)
9. Oral nutritional supplements can reduce the complications, mortality, length of hospital stays, and the probability of re-hospitalization.	38 (8.70)	130 (29.75)	269 (61.56)
10. The use of oral nutritional supplements can save patients’ total medical costs.	33 (7.55)	98 (22.43)	306 (70.02)

The median attitude score was 34 (30, 39), corresponding to an unfavorable attitude. Although 45.54% of participants were willing to try oral nutritional supplements to alleviate the nutritional problems during radiotherapy for HNC (question A5), 45.31% remained neutral on the topic, 7.55% disagreed, and 1.6% strongly disagreed. However, more than half of the participants expressed concern about the high price of oral nutritional supplements, describing it as a heavy economic burden (question A10: 42.79% agreed and 15.33% strongly agreed) (Table 3).

**Table 3:** Distribution of scores in the attitude dimension.

*N* (%)	Strongly agree	Agree	Neutral	Disagree	Strongly disagree
1. Thinking that the tumor and subsequent treatment affected their eating habits.	50 (11.44)	165 (37.76)	189 (43.25)	25 (5.72)	8 (1.83)
2. Feeling malnourished and in need of better nutrition.	49 (11.21)	146 (33.41)	184 (42.11)	48 (10.98)	10 (2.29)
3. Thinking that additional nutritional supplements are necessary for general cancer patients.	61 (13.96)	200 (45.77)	156 (35.7)	14 (3.2)	6 (1.37)
4. Thinking that oral nutritional supplements are an effective way to improve nutritional status.	48 (10.98)	174 (39.82)	192 (43.94)	17 (3.89)	6 (1.37)
5. Would like to try oral nutritional supplements to alleviate the nutritional problems during radiotherapy for head-and-neck cancer.	46 (10.53)	153 (35.01)	198 (45.31)	33 (7.55)	7 (1.6)
6. Thinking that oral nutritional supplements are more convenient and safe than other nutritional supplement methods.	48 (10.98)	175 (40.05)	189 (43.25)	21 (4.81)	4 (0.92)
7. Believing that the effectiveness of oral nutritional supplementation varies from person to person and may not be appropriate for all patients with head-and-neck cancer.	40 (9.15)	153 (35.01)	213 (48.74)	25 (5.72)	6 (1.37)
8. Thinking that there may be adverse reactions or excessive risks of oral nutritional supplements, and the safety needs to be considered.	36 (8.24)	131 (29.98)	214 (48.97)	50 (11.44)	6 (1.37)
9. Concerned that some components of oral supplements may interact with other medications.	35 (8.01)	141 (32.27)	219 (50.11)	37 (8.47)	5 (1.14)
10. Worried about the high price of oral nutritional supplements, which are not covered by medical insurance, and the heavy economic burden.	67 (15.33)	187 (42.79)	153 (35.01)	24 (5.49)	6 (1.37)

The median practice score was 15 (13, 17), corresponding to proactive behavior. The majority of respondents (84.67%) reported always or often paying attention to adequate nutritional intake (question P2), but approximately 18.53% of participants rarely or never wanted to learn more about the topic (question P4) (Table 4).

**Table 4:** Distribution of scores in the practice dimensions.

*N* (%)	Always	Often	Sometimes	Rarely	Never
1. Following doctor’s advice for regular radiotherapy and chemotherapy.	333 (76.20)	70 (16.02)	18 (4.12)	8 (1.83)	8 (1.83)
2. Paying attention to strengthening the intake of adequate nutrition in daily life, such as diversifying food and ensuring the intake of protein foods.	201 (46.00)	169 (38.67)	52 (11.90)	11 (2.52)	4 (0.92)
3. Using or having used oral nutritional supplements.	70 (16.02)	62 (14.19)	80 (18.31)	103 (23.57)	122 (27.92)
4. Having a desire to learn more about nutritional supplements.	114 (26.09)	133 (30.43)	109 (24.94)	56 (12.81)	25 (5.72)

### Correlation analysis among KAP scores

Correlation analysis demonstrated that knowledge score was positively correlated with attitude score (r = 0.379, *p* < 0.001) and practice score (r = 0.395, *p* < 0.001); moreover, attitude and practice scores were also positively correlated (r = 0.363, *p* < 0.001) (Table 5).

**Table 5:** Correlations between knowledge, attitude, and practice scores.

Dimension	Knowledge	Attitude	Practice
Knowledge	1		
Attitude	0.379 (*p* < 0.001)	1	
Practice	0.395 (*p* < 0.001)	0.363 (*p* < 0.001)	1

### SEM analysis

**Figure 1:**
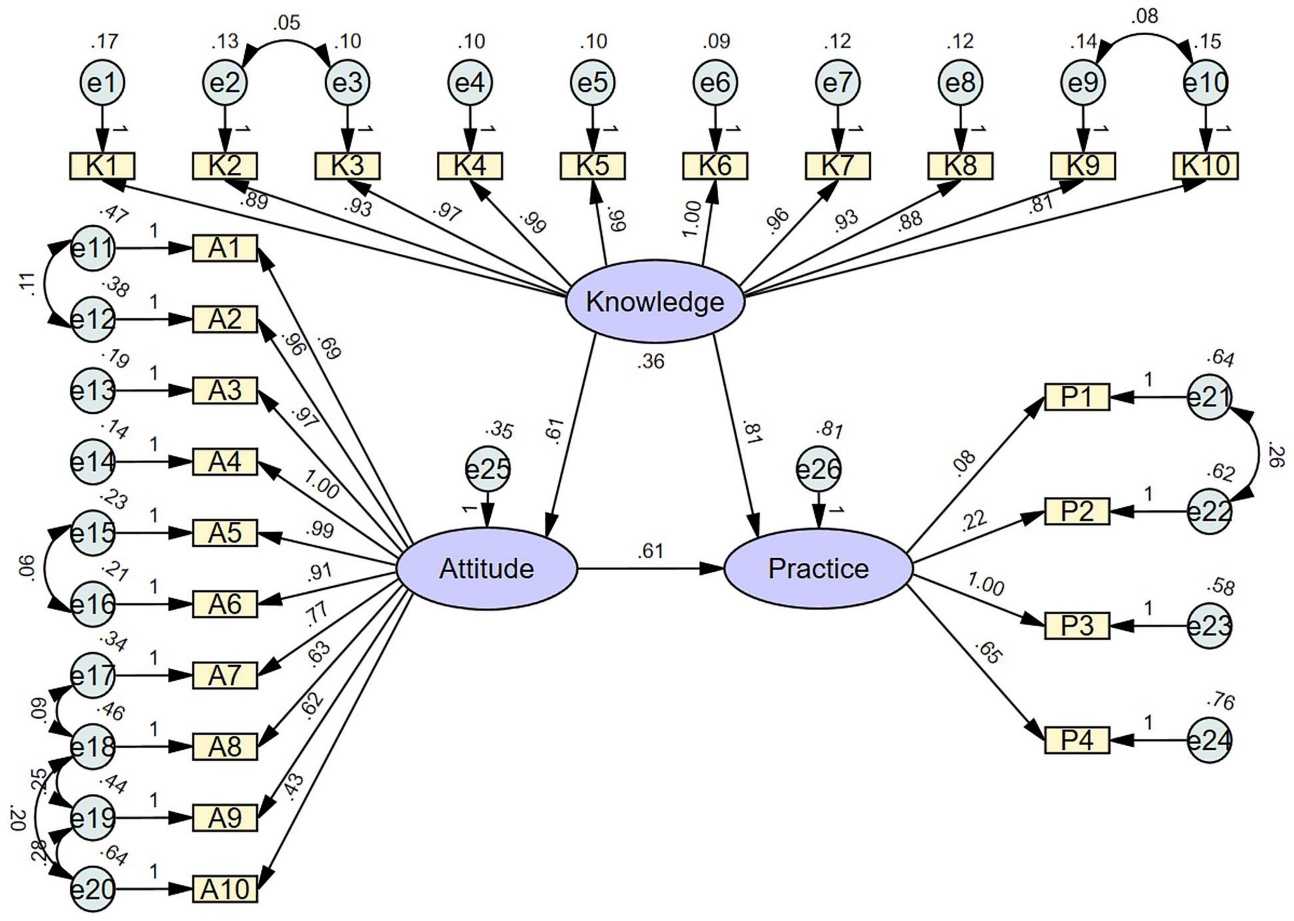
Structural equation model (SEM) depicting the relationships among the latent variables: knowledge, attitude, and practice. Purple ellipses represent latent variables (unobserved constructs), including overall knowledge, attitude, and practice. Yellow rectangles represent observed variables (measured questionnaire items) that serve as indicators of the latent variables. Light blue circles represent error terms or residuals associated with the observed variables. Single-headed arrows indicate hypothesized directional relationships (pathways) between latent variables and from latent variables to observed variables, with standardized path coefficients labeled on the arrows.

SEM was used to further explore the influence of knowledge on practice, with model fit indices corresponding to the acceptable model fit (Supplementary Table S1). As demonstrated in Figure 1 and Supplementary Table S2, it was found that knowledge had a direct positive effect on attitude (*β* = 0.613, *p* < 0.001) and on practice (β = 0.807, *p* < 0.001), while attitude had a direct effect on practice (β = 0.614, *p* < 0.001).

## Discussion

The study found that knowledge about oral nutritional supplements among participants was poor, with an unclear understanding of their benefits and proper use, while attitudes were mostly neutral, and concerns about cost were prevalent. Despite this finding, the majority of participants reported proactive behavior toward ensuring adequate nutrition, though a notable minority showed limited interest in learning more. The positive associations between KAP dimensions and differences in scores between participants who reportedly participated in hospital education strongly suggest that the provision of personalized educational initiatives could be effective in the studied population. Improving awareness of oral nutritional supplements and their advantages would help to decrease complications for HNC patients following radiotherapy.

Insufficient nutritional knowledge is a common issue among Chinese cancer patients, as discussed in the most recent systematic review (14) and confirmed by the results of our study. Additionally, in the present study, only 27.0% of participants reported that they participated in educational interventions about oral nutritional supplements, while 18.53% rarely or never wanted to learn more about the topic. Although participants who received educational information had significantly higher scores for each KAP dimension, the overall low proportion highlights the urgent need for targeted education. Both correlation analysis and SEM in this study confirmed notable associations between KAP dimensions, highlighting a significant role of knowledge in shaping attitudes and practices; in-hospital education could, therefore, provide HNC patients with clear, personalized guidance on the benefits, proper use, and timing of dietary supplements, addressing common misconceptions and building confidence in their effectiveness (22).

A key strength of the study is its inclusion of a diverse population, enhancing the reliability and generalizability of the findings and allowing the identification of specific subgroups that require special attention. Previous studies often reported that KAP differs significantly between urban and rural residents due to disparities in access to education, healthcare resources, and exposure to health information (23). Urban residents typically have higher knowledge and more proactive health practices, while rural residents may face barriers such as limited healthcare access and cultural factors that influence attitudes and behaviors (24). Although the majority of participants in the present study were from urban areas, the subpopulation of rural residents was 49.20%, presenting almost equal distribution, which allowed for better comparison of KAP. It was found that the knowledge scores were significantly higher in urban residents, in line with previous studies, but attitude and practice scores did not differ. One of the controversial findings is that patients with poor quality of sleep (29.52% of the entire population) had significantly higher attitude scores, as well as slightly higher knowledge scores. This could be at least partly explained by the fact that KAP scores did not differ according to the time of diagnosis but were all significantly higher in patients who underwent more radiotherapy treatments, especially in the knowledge domain. These patients might have been both exhausted by the disease and had a better understanding of its management.

It was previously shown that, by offering tailored support and resources, such education can empower patients to incorporate supplements into their daily routines, improving adherence and optimizing their nutritional status during treatment (25, 26). In the present study, practice scores were generally higher, suggesting that, despite insufficient knowledge, participants were ready to adopt oral nutritional supplements. Notably, practice scores were lower in patients with laryngeal/pharyngeal cancer, indicating the need for a more proactive approach to patient education, given the unique challenges associated with these diagnoses. In addition, KAP scores differed significantly with the household income, while more than half of the participants expressed worry about the high price of oral nutritional supplements, describing it as a heavy economic burden. Economic barriers significantly limit cancer patients’ access to essential nutritional resources, including oral nutritional supplements, leading to suboptimal dietary intake and poorer health outcomes (27, 28). High costs can force patients to prioritize other medical expenses, compromising their ability to meet dietary needs critical for supporting treatment and recovery (29). These financial constraints often exacerbate malnutrition and reduce the effectiveness of treatment, highlighting the importance of cost-reduction measures and support programs for vulnerable patients (30). The above concerns suggest the need for cost-reduction strategies, such as subsidies, insurance coverage, or cost-effective alternatives, to increase accessibility and adherence among patients.

This study has several limitations that should be considered when interpreting the results. First, data were collected only from several hospitals in northern China, which may limit the generalizability of the findings due to regional differences in culture, socioeconomic status, or healthcare contexts. Second, although the sample size was relatively big, some of the subgroup analyses may have had reduced statistical power and an increased risk of bias. Third, the cross-sectional nature of the study captures data at a single point in time, preventing analysis of changes in knowledge, attitudes, and practices over time. Fourth, practical aspects such as the impact of different educational interventions, patient adherence to recommendations, and long-term outcomes were not analyzed, limiting the study’s ability to provide actionable strategies for improving patient care. Fifth, the study population comprised both newly diagnosed and recurrent/metastatic HNC patients. Differences in disease status may affect patients’ psychological state and responses, potentially limiting data comparability. Finally, this study included patients at various stages of their oncologic treatment, which may have affected their responses. Treatment-related adverse effects, such as fatigue, pain, or emotional distress, could have influenced patients’ mood and motivation, potentially biasing their answers. Future research should address these limitations by employing longitudinal study designs with larger and more diverse populations, stratifying analyses according to disease stage, and controlling for treatment timing or focusing on more homogeneous patient cohorts to minimize potential bias.

In conclusion, our findings indicate that patients with HNC undergoing radiotherapy possess poor knowledge and exhibit unfavorable attitudes regarding oral nutritional supplements, despite demonstrating proactive practices. These findings highlight the importance of implementing targeted education programs to improve awareness of supplements, their advantages, and their role in minimizing complications for HNC patients following radiotherapy. Considering the positive correlation between knowledge, attitudes, and practices, the provision of personalized medical education is essential for effective disease management.

## Data Availability

The original contributions presented in the study are included in the article/Supplementary material, further inquiries can be directed to the corresponding authors.

## References

[1] Gormley M Creaney G Schache A Ingarfield K Conway DI. Reviewing the epidemiology of head and neck Cancer: definitions, trends and risk factors. Br Dent J. (2022) 233:780–6. doi: 10.1038/s41415-022-5166-x, PMID: 36369568 PMC9652141

[2] Toft K McLachlan K Winton M Mactier K Hare N Nugent C. Global assessment of swallow function (Gasf) following Vmat radiotherapy for head and neck squamous cell carcinoma. Tech Innov Patient Supp Radiat Oncol. (2024) 32:100272. doi: 10.1016/j.tipsro.2024.100272, PMID: 39346655 PMC11439550

[3] Vergauwen A Van den Steen L Baudelet M Van Nuffelen G. Head and neck cancer survivors' assessment of mealtimes: translation and validation: assessment and rehabilitation of dysphagia in head and neck cancer patients. Dysphagia. (2024) 40:737–46. doi: 10.1007/s00455-024-10771-6, PMID: 39433565 PMC12328494

[4] Miller EM Walters RK Nguyen SA Harper JL Depaoli B O'Rourke AK. Time to onset of dysphagia following head and neck radiation. Dysphagia. (2024) 40:841–50. doi: 10.1007/s00455-024-10782-3 39540921 PMC12328464

[5] Gómez Á García-Chabur MA Peñaranda D Gómez-Mendoza A Forero JC. Chemotherapy/radiotherapy-induced dysphagia in head and neck tumors: a challenge for otolaryngologists in low- to middle-income countries. Dysphagia. (2024) 40:515–27. doi: 10.1007/s00455-024-10756-5, PMID: 39317843 PMC12145316

[6] Abouegylah M Udugamasooriya SS Ahmed AA Tas KT Lishewski P Smalec E. The role of oral nutritional supplements in head and neck cancer patients undergoing chemoradiotherapy. Healthcare. (2024) 12:2070. doi: 10.3390/healthcare12202070, PMID: 39451484 PMC11506854

[7] Lili Z Miao H He Y Tao W Xuejun X. Effects of oral nutritional supplements on nutritional status and toxicity of hospitalized patients with head and neck tumors during chemoradiotherapy. J Cancer Control Treatment. (2020) 33:858–65.

[8] Cereda E Cappello S Colombo S Klersy C Imarisio I Turri A. Nutritional counseling with or without systematic use of Oral nutritional supplements in head and neck Cancer patients undergoing radiotherapy. Radiother Oncol. (2018) 126:81–8. doi: 10.1016/j.radonc.2017.10.015, PMID: 29111172

[9] Bozzetti F Cotogni P. Nutritional issues in head and neck cancer patients. Healthcare. (2020) 8:102. doi: 10.3390/healthcare8020102, PMID: 32316416 PMC7348698

[10] de Oliveira Faria S Simões Lima GA Lopes Carvalho A Nader Marta G Howell D Eluf-Neto J. Clinically significant changes in health-related quality of life in head and neck cancer patients following intensive nutritional care during radiotherapy. Eur J Oncol Nurs. (2022) 56:102065. doi: 10.1016/j.ejon.2021.102065, PMID: 34826722

[11] Dai T Xian J Li X Wang Z Hu W. Effect of nutrition impact symptoms on Oral nutritional supplements energy intake and use days in patients with head and neck Cancer: a cross-sectional study. Cancer Med. (2024) 13:e7288. doi: 10.1002/cam4.7288, PMID: 38770538 PMC11106646

[12] Andrade C Menon V Ameen S Kumar PS. Designing and conducting knowledge, attitude, and practice surveys in psychiatry: practical guidance. Indian J Psychol Med. (2020) 42:478–81. doi: 10.1177/0253717620946111, PMID: 33414597 PMC7750837

[13] Santesso N Akl E Bhandari M Busse JW Cook DJ Greenhalgh T. A practical guide for using a survey about attitudes and behaviors to inform health care decisions. J Clin Epidemiol. (2020) 128:93–100. doi: 10.1016/j.jclinepi.2019.11.020, PMID: 32987165

[14] Tang H Zhang Y Cao B Liang Y Na R Yang Z. Knowledge, attitudes and behaviors toward healthy eating among Chinese Cancer patients treated with chemotherapy: a systematic review. Asia Pac J Oncol Nurs. (2023) 10:100163. doi: 10.1016/j.apjon.2022.100163, PMID: 36471827 PMC9718976

[15] Alberda C Alvadj-Korenic T Mayan M Gramlich L. Nutrition Care in Patients with head and neck or esophageal Cancer: the patient perspective. Nutr Clin Pract. (2017) 32:664–74. doi: 10.1177/0884533617725050, PMID: 28841392

[16] Mayre-Chilton K Talwar B Goff L. Different experiences and perspectives between head and neck cancer patients and their care-givers on their daily impact of a gastrostomy tube. J Hum Nutr Diet. (2011) 24:449–59. doi: 10.1111/j.1365-277X.2011.01165.x, PMID: 21649745

[17] Branch CPAROTP, Branch CMAROT, Committee. CA-CATRTP. Radiation therapy guidelines for head and neck tumors in China (2021 edition). Int J Oncol. (2022) 2:65–72. doi: 10.3760/cma.j.cn371439-20210831-00010

[18] Association ROSotCM. Expert consensus on Oral nutritional supplementation for radiation therapy patients with oncology (2017). Chin J Radiat Oncol. (2017) 11:1239–47. doi: 10.3760/cma.j.issn.1004-4221.2017.11.001

[19] Pan XF Wang L Pan A. Epidemiology and determinants of obesity in China. Lancet Diabetes Endocrinol. (2021) 9:373–92. doi: 10.1016/s2213-8587(21)00045-0, PMID: 34022156

[20] Shao XX Fang LY Guo XR Wang WZ Shi RX Lin DP. Knowledge, attitude, and practice of patients living with inflammatory bowel disease: a cross-sectional study. World J Gastroenterol. (2023) 29:5818–33. doi: 10.3748/wjg.v29.i43.5818, PMID: 38074915 PMC10701310

[21] Gunawan J Marzilli C Aungsuroch Y. Establishing appropriate sample size for developing and validating a questionnaire in nursing research. Belitung Nurs J. (2021) 7:356–60. doi: 10.33546/bnj.1927, PMID: 37496511 PMC10367972

[22] Shinn EH Garden AS Chen M Basen-Engquist K Fellman B Hutcheson K. Self-management intervention improves patient adherence to swallowing exercises during radiation for head and neck Cancer. Head Neck. (2024) 46:2878–89. doi: 10.1002/hed.27832, PMID: 38873861 PMC11801331

[23] Fu L Shi Y Li S Jiang K Zhang L Wen Y. Healthy diet-related knowledge, attitude, and practice (Kap) and related socio-demographic characteristics among middle-aged and older adults: a cross-sectional survey in Southwest China. Nutrients. (2024) 16:869. doi: 10.3390/nu16060869, PMID: 38542780 PMC10974890

[24] Kim S Kim S. Analysis of the impact of health beliefs and resource factors on preventive behaviors against the Covid-19 pandemic. Int J Environ Res Public Health. (2020) 17:8666. doi: 10.3390/ijerph17228666, PMID: 33266386 PMC7700576

[25] Frenkel M Morse MB Narayanan S. Addressing patient requests to add dietary supplements to their Cancer care-a suggested approach. Nutrients. (2023) 15:5029. doi: 10.3390/nu15245029, PMID: 38140288 PMC10745580

[26] Lacey J Huston A Lopez G Vozmediano JR Lam CS Narayanan S. Establishing an integrative oncology service: essential aspects of program development. Curr Oncol Rep. (2024) 26:200–11. doi: 10.1007/s11912-024-01504-x, PMID: 38358637

[27] Nasrah R Van Der Borch C Kanbalian M Jagoe RT. Defining barriers to implementation of nutritional advice in patients with Cachexia. J Cachexia Sarcopenia Muscle. (2020) 11:69–78. doi: 10.1002/jcsm.12490, PMID: 31436033 PMC7015253

[28] Beichmann B Henriksen C Paur I Paulsen MM. Barriers and facilitators of improved nutritional support for patients newly diagnosed with Cancer: a pre-implementation study. BMC Health Serv Res. (2024) 24:815. doi: 10.1186/s12913-024-11288-2, PMID: 39010098 PMC11251100

[29] Victor MT Zheng W Park SJ Jiang SIB Guo TW. Insurance status is associated with recurrence in cutaneous head and neck squamous cell carcinoma. Otolaryngolo Head Neck Surg. (2024) 170:132–40. doi: 10.1002/ohn.504, PMID: 37622529

[30] Barnes JM Johnston KJ Johnson KJ Chino F Osazuwa-Peters N. State public assistance spending and survival among adults with Cancer. JAMA Netw Open. (2023) 6:e2332353. doi: 10.1001/jamanetworkopen.2023.32353, PMID: 37669050 PMC10481229

