# Supplementary material for: Knowledge, attitude, and practice of oral nutritional supplements in patients undergoing radiotherapy for head-and-neck cancer
Source: Front Nutr. 2025 Oct 22;12:1633423. doi: 10.3389/fnut.2025.1633423 (PMC12586067; doi:10.3389/fnut.2025.1633423)
Supplement: Supplementary file 1 [file Table_1.DOCX]

**Table S1. Model Fit indices for Structural Equation Model**

| **Indicators** | **Reference** | **Actual** |
| --- | --- | --- |
| CMIN/DF | 1-3: Excellent, 3-5: Good | 2.498 |
| RMSEA | <0.08: Good | 0.059 |
| IFI | >0.8: Good | 0.955 |
| TLI | >0.8: Good | 0.948 |
| CFI | >0.8: Good | 0.955 |

RMSEA – root mean square error of approximation; SRMR – standardized root mean squared residual; CFI – comparative fit index; TLI – Tucker-Lewis index AGFI – Adjusted goodness-of-fit.

**Table S2. Standardized Path Coefficients, Standard Errors, and Significance Levels of the Structural Equation Model**

| **Path** |  |  | **Estimate** | **S.E.** | **C.R.** | **P** |
| --- | --- | --- | --- | --- | --- | --- |
| Attitude | <--- | Knowledge | 0.613 | 0.055 | 11.12 | *** |
| Practice | <--- | Attitude | 0.614 | 0.098 | 6.243 | *** |
| Practice | <--- | Knowledge | 0.807 | 0.113 | 7.155 | *** |
| K1 | <--- | Knowledge | 0.89 | 0.04 | 21.987 | *** |
| K2 | <--- | Knowledge | 0.93 | 0.038 | 24.485 | *** |
| K3 | <--- | Knowledge | 0.972 | 0.036 | 27.023 | *** |
| K4 | <--- | Knowledge | 0.989 | 0.036 | 27.244 | *** |
| K5 | <--- | Knowledge | 0.991 | 0.036 | 27.55 | *** |
| K6 | <--- | Knowledge | 1 |  |  |  |
| K7 | <--- | Knowledge | 0.958 | 0.037 | 25.848 | *** |
| K8 | <--- | Knowledge | 0.935 | 0.037 | 25.414 | *** |
| K9 | <--- | Knowledge | 0.882 | 0.038 | 23.322 | *** |
| K10 | <--- | Knowledge | 0.81 | 0.037 | 21.65 | *** |
| A10 | <--- | Attitude | 0.426 | 0.058 | 7.34 | *** |
| A9 | <--- | Attitude | 0.624 | 0.05 | 12.363 | *** |
| A8 | <--- | Attitude | 0.626 | 0.051 | 12.206 | *** |
| A7 | <--- | Attitude | 0.769 | 0.047 | 16.456 | *** |
| A6 | <--- | Attitude | 0.914 | 0.042 | 21.791 | *** |
| A5 | <--- | Attitude | 0.986 | 0.044 | 22.16 | *** |
| A4 | <--- | Attitude | 1 |  |  |  |
| A3 | <--- | Attitude | 0.966 | 0.041 | 23.353 | *** |
| A2 | <--- | Attitude | 0.955 | 0.051 | 18.613 | *** |
| A1 | <--- | Attitude | 0.687 | 0.053 | 13.014 | *** |
| P1 | <--- | Practice | 0.082 | 0.036 | 2.282 | 0.022 |
| P2 | <--- | Practice | 0.218 | 0.037 | 5.821 | *** |
| P3 | <--- | Practice | 1 |  |  |  |
| P4 | <--- | Practice | 0.648 | 0.058 | 11.151 | *** |

Note: This table presents the standardized estimates from the structural equation modeling (SEM) analysis, including latent-to-latent and latent-to-observed variable paths. Estimate indicates the standardized path coefficient; S.E. represents the standard error; C.R. is the critical ratio (Estimate/S.E.); and P denotes the statistical significance. Asterisks (***) indicate p < 0.001.
